# Gut metagenomic and short chain fatty acids signature in hypertension: a cross-sectional study

**DOI:** 10.1038/s41598-020-63475-w

**Published:** 2020-04-15

**Authors:** Lorena Calderón-Pérez, Maria José Gosalbes, Silvia Yuste, Rosa M. Valls, Anna Pedret, Elisabet Llauradó, Nuria Jimenez-Hernandez, Alejandro Artacho, Laura Pla-Pagà, Judit Companys, Iziar Ludwig, Maria-Paz Romero, Laura Rubió, Rosa Solà

**Affiliations:** 1 Eurecat, Centre Tecnològic de Catalunya, Unitat de Nutrició i Salut, Reus, Spain; 2 Universitat Rovira i Virgili, Facultat de Medicina i Ciències de la Salut, Functional Nutrition, Oxidation, and Cardiovascular Diseases Group (NFOC-Salut), C/Sant Llorenç 21, 43201 Reus, Spain; 3 Fundación para el Fomento de la Investigación Sanitaria y Biomédica, Valencia, Spain; 4 CIBER en Epidemiología y Salud Pública (CIBEResp), Madrid, Spain; 5 Food Technology Department, XaRTA-TPV, Agrotecnio Center, Escola Tècnica Superior d’Enginyeria Agrària, University of Lleida, Avda/Alcalde Rovira Roure 191, 25198 Lleida, Catalonia Spain; 6 Hospital Universitari Sant Joan de Reus, Reus, Spain

**Keywords:** Predictive markers, Hypertension

## Abstract

Hypertension is an independent and preventable risk factor for the development of cardiovascular diseases, however, little is known about the impact of gut microbiota composition in its development. We carried out comprehensive gut microbiota analysis and targeted metabolomics in a cross-sectional study of 29 non-treated hypertensive (HT) and 32 normotensive (NT) subjects. We determined fecal microbiota composition by 16S rRNA gene sequencing and bacterial functions by metagenomic analysis. The microbial metabolites analysed were short chain fatty acids (SCFA) both in plasma and feces, and trimethylamine N-oxide (TMAO) in plasma. The overall bacterial composition and diversity of bacterial community in the two groups were not significantly different. However, Ruminococcaceae NK4A214, Ruminococcaceae_UCG-010, Christensenellaceae_R-7*, Faecalibacterium prausnitzii* and *Roseburia hominis* were found to be significantly enriched in NT group, whereas*, Bacteroides coprocola, Bacteroides plebeius* and genera of *Lachnospiraceae* were increased in HT patients. We found a positive correlation between the HT-associated species and systolic and diastolic blood pressure after adjusted for measured confounders. SCFA showed antagonistic results in plasma and feces, detecting in HT subjects significant higher levels in feces and lower levels in plasma, which could indicate a less efficient SCFA absorption. Overall, our results present a disease classifier based on microbiota and bacterial metabolites to discriminate HT individuals from NT controls in a first disease grade prior to drug treatment.

## Introduction

Hypertension is one of the leading risk factors for the development of cardiovascular diseases (CVD) globally, and lifestyle measures are effective as a preventive strategies^1^. In 2015, hypertension based on office blood pressure (BP) reported the highest rates with a global prevalence of 1.13 billion cases, and 150 million in central and eastern Europe regardless of the income countries status^2–4^.

The identification of the determinants of hypertension is still challenging although it is well recognized its multifactorial etiology^5^. It has been elucidated the interplay of genetic and environmental factors related with risk-conferring behaviors, such as, smoking, lack of physical activity, alcohol consumption and unhealthy diet^6,7^.

Recently, it has been evidenced the role of gut microbiota dysbiosis on the modulation of high BP, both in animal and human hypertension^8^. Mell *et al*. (2015) were the first to demonstrate the differential fecal microbial composition of Dahl salt-sensitive and Dahl salt-resistant rats^9^. In the same period, Yang *et al*. (2015) also found a clear gut dysbiosis in spontaneously hypertensive rats (SHR) mediated by a decrease in microbial richness and diversity compared to the normotensive Wistar-Kyoto rats^8^. In line with these results, changes in gut microbiota composition also occur in humans at different hypertension grades, and high BP appears to be transferrable from hypertensive donor to germ-free mice through fecal microbiota transplantation^10^. It was also recently reported that, these changes in the composition of SHR rats gut microbiota seems to be at least partially associated with altered integrity of the gut epithelial barrier and altered inflammatory status^11^.

Changes in gut bacteria composition precede alterations in the level of metabolic microbiota-derived end products in the bloodstream. A clear example of bacterial metabolites are short chain fatty acids (SCFA) derived from dietary fibers fermentation, being the most abundant butyrate, acetate and propionate^12^. The low abundance of SCFA-producing bacteria in SHR rats disrupt the gut bacterial balance leading to immunological, physiological and metabolic alterations in the host status that can influence BP homeostasis^8^. Although the mechanism by means SCFA regulates BP has not been fully studied, Pluznick *et al*. (2013) showed how BP can be modulated in response to circulating SCFA via G-protein-coupled receptors (GPCRs) expression in the vascular or renal tissues^13,14^.

Studies are conflicting as to whether SCFA are beneficial or detrimental to cardiometabolic health. In a recent study de la Cuesta-Zuluaga *et al*. (2018)^15^ reported in a human cohort that higher fecal SCFA concentrations were associated with a measure of gut permeability and also hypertension. The same authors also highlighted that more studies are needed analyzing both fecal and circulating SCFA in humans to test the hypothesis that the association of higher fecal SCFA with cardiometabolic dysregulation is due to less efficient SCFA absorption to circulation.

In addition to SCFA, other bacterial metabolites such as trimethylamine N-oxide (TMAO) might also be involved in the pathogenesis of CVD. TMAO is derived from dietary sources of phosphatidylcholine (lecithin), such as red meat, dairy products, eggs and fish^16,17^. Elevated TMAO plasma levels are associated with high atherosclerosis burden, and specific bacterial genera have been identified as a putative choline degraders and TMAO producers^18–20^. However, the existing body of literature is sparse regarding TMAO and hypertension.

To address the questions raised above, we carried out a comprehensive taxonomic and functional analysis and targeted metabolomics in a cross-sectional study of 29 non-treated grade I hypertensive (HT) and 32 normotensive (NT) subjects, with special emphasis on targeting fecal metabolites that appear to be a novel target for hypertension treatment.

## Results and Discussion

### Clinical and lifestyle characteristics of the study participants

From 80 subjects who were assessed for eligibility, 19 were excluded for not meeting the inclusion criteria for systolic blood pressure (SBP). The remaining 61 participants were assigned to HT group (n = 29) and NT control group (n = 32) (**Additional file 1:** Fig. S1). Anthropometric and clinical characteristics of the total study population segregated by HT and NT, are shown in Table 1. A total of 10 females and 19 males with a mean age of 53.7 years were assigned to HT group, and 16 females and 16 males with a mean age of 41.1 years were designated into the NT group. Although age was significantly different between HT and NT (p < 0.001), both groups were in the same range of age (adulthood).

**Table 1:** Baseline characteristics of participants.

Variable	Hypertensive (n = 29)	Normotensive (n = 32)	p-value
Age, *y*	53.7 ± 9.6	41.1 ± 9.1	<0.001
Gender, *(F/M)*	(10/19)	(16/16)	0.301
SBP, *mm Hg*	153.1 ± 14.6	109.7 ± 7.1	<0.001
DBP, *mm Hg*	91.0 ± 8.8	65.7 ± 6.7	<0.001
Weight, *kg*	75.3 ± 9.3	68.9 ± 10.8	0.017
BMI, *k*g/m^2^	26.2 ± 2.5	23.8 ± 2.7	<0.001
Waist circumference, *cm*	94.4 ± 8.3	84.0 ± 9.0	<0.001
Fat mass, *%*	26.6 ± 7.9	22.1 ± 7.8	0.037
FBG, *mg/dL*	91.2 ± 11.3	81.1 ± 7.5	0.001
Cholesterol, *mg/dL*			
Total	199.6 ± 43.9	181.7 ± 34.7	0.017
LDL	123.7 ± 21.3	100.7 ± 33.2	0.002
HDL	62.6 ± 14.0	64.9 ± 18.0	0.580
Triglycerids, *mg/dL*	97.3 ± 38.8	80.7 ± 42.6	0.067
Physical activity, *%*			0.729
Inactive	6.9	0.0	
Very low activity	10.3	10.0	
Low activity	10.3	6.7	
Moderate activity	20.7	20.0	
High activity	51.7	63.3	
Sleep Quality, *%*			0.299
Good quality	48.3	63.3	
Poor quality	51.7	36.7	

Data expressed as mean ± standard deviation or percentage. SBP, systolic blood pressure; DBP, diastolic blood pressure; BMI, body mass index; FBG, fasting blood glucose; LDL. low density lipoproteins; HDL, high density lipoproteins. P-value for gender, physical activity and sleep quality was calculated by Fisher’s exact test. P-value for age, SBP, DBP, weight, BMI, waist circumference, fat mass, FBG, cholesterol and triglycerides was calculated by Student’s t-test and Mann-Whitney U test.

 Significant differences between groups were observed in anthropometric parameters as weight (HT, 75.3 ± 9.3 kg; NT, 68.9 ± 10.8 kg; p = 0.017), body mass index (BMI) (HT, 26.2 ± 2.5 kg/m^2^; NT, 23.8 ± 2.7 kg/m^2^; p < 0.001), waist circumference (HT, 94.4 ± 8.3 cm; NT, 84.0 ± 9.0 cm; p < 0.001) and fat mass (HT, 26.6 ± 7.9%; NT: 22.1 ± 7.8%; p = 0.037). Results from analytical parameters showed that HT group had higher levels of fasting blood glucose (HT, 91.2 ± 11.3 mg/dL; NT, 81.1 ± 7.5 mg/dL; p = 0.001), total cholesterol (HT, 199.6 ± 43.9 mg/dL; NT, 181.7 ± 34.7 mg/dL; p = 0.017) and low density lipoprotein (LDL)-cholesterol (HT, 123.7 ± 21.3 mg/dL; NT, 100.7 ± 33.2 mg/dL; p = 0.002) than NT group. Despite these differences between groups, it is important to highlight that all baseline clinical parameters remained within normal values except for SBP (HT, 151.8 ± 16.2 mmHg; NT, 109.7 ± 7.1 mmHg; p < 0.001) and DBP (HT, 90.2 ± 9.9 mmHg; NT, 65.7 ± 6.7 mmHg; p < 0.001). Previous evidence suggest that gut microbial composition appears to be altered with lifestyle, obesity and cardiometabolic disease^21^. From our results, the differences found in clinical parameters between HT and NT remained among healthy ranges. So, we hypothesize that microbial changes might be mainly related to hypertension, and the other parameters would not contribute to the changes.

No differences were found in physical activity and sleep quality. Diet composition was similar between groups with exception of total dietary fiber intake, which was greater in the NT group (HT, 20.95 ± 9.90 g; NT, 25.90 ± 11.63 g; p = 0.029) (**Additional file 2:** Table S1). Reported data from food frequency questionnaire (FFQ) showed differences between HT and NT groups in daily mean intake of processed meat products (p = 0.016), potatoes (p = 0.05), natural juices (p = 0.008) and coffee (p = 0.033) showing a higher consumption in HT patients. The whole-grain cereals intake was higher in NT group (p = 0.034) (**Additional file 3:** Table S2).

Subjects taking antihypertensive drugs were excluded in order to assess patients in a first disease grade prior to drug treatment and to avoid any drug interference on gut microbiome. It should be noted that 4 NT and 6 HT subjects were taking occasionally other drugs, specifically, analgesic and nonsteroidal anti-inflammatory (NSAIDs), with the last intake at least 30 days before the inclusion visit. Although changes in the composition and biodiversity of the intestinal microbiome have been described after usual NSAIDs administration^22^, none of the study participants were chronic NSAIDs users.

### Microbiota composition analysis

**Figure 1:**
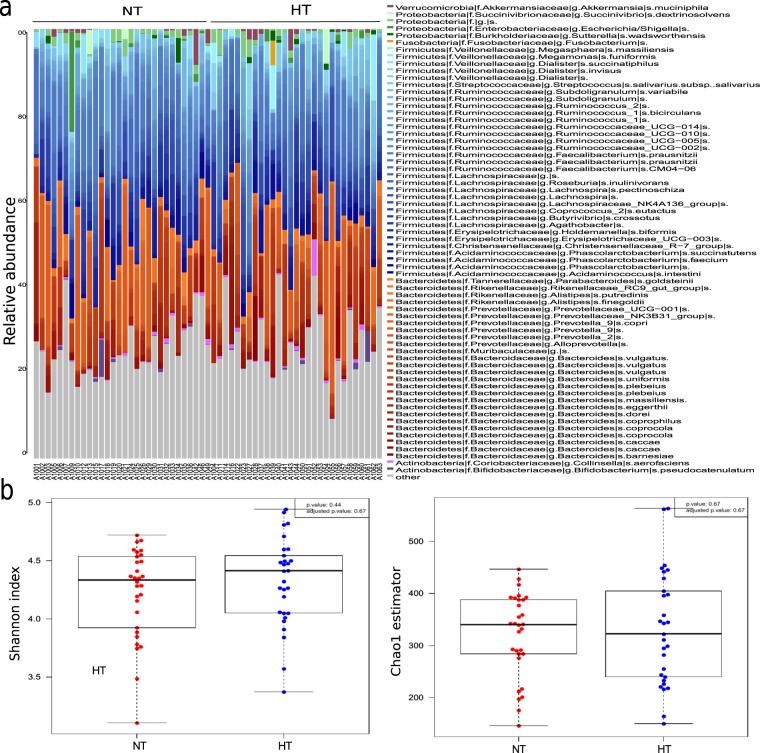
Difference of gut microbial community between hypertensive (HT) and normotensive (NT) groups. **(a)** Bacterial composition between groups at ASV level. **(b)** Differences between groups in diversity (Shanon index) and richness (Chao1 index) of bacterial community.

We obtained 9,609,446 amplicon raw sequences for 59 fecal samples that we filtered by length, quality and chimera giving a total of 7,171,741 filtered sequences with an average of 129,125 sequences per sample in HT and 114,237 sequences per sample in NT. We used the DADA2 pipeline generating 4491 amplicon sequence variants (ASV) available for further analysis. Taxonomic assignment was performed at ASV and genus level. Both groups, NT and HT, presented Firmicutes (45.94% and 45.98%, respectively) and Bacteroidetes (25.62% and 26.35%, respectively) as the major two phyla. Also, Proteobacteria (1.42% and 0.83%) and Actinobacteria (0.98% and 1.2%) were detected in NT and HT groups (Fig. 1a). In Firmicutes phylum, we found that *Faecalibacterium* (12.1% and 9.7%), *Ruminococcus* (3.1% and 1.9%), *Lachnospira* (2.68% and 2.46%), *Phascolarctobacterium* (2.5% and 2.81%), *Roseburia* (5.14% and 4.17%) and *Dialister* (1.05% and 1.01%) were the main genera in NT and HT. For Bacteroidetes phylum, *Bacteroides* (12% and 13.45%), *Prevotella* (9.1% and 8.2%) and *Alistipes* (1.8% and 1.2%) were the most abundant taxa.

**Figure 2:**
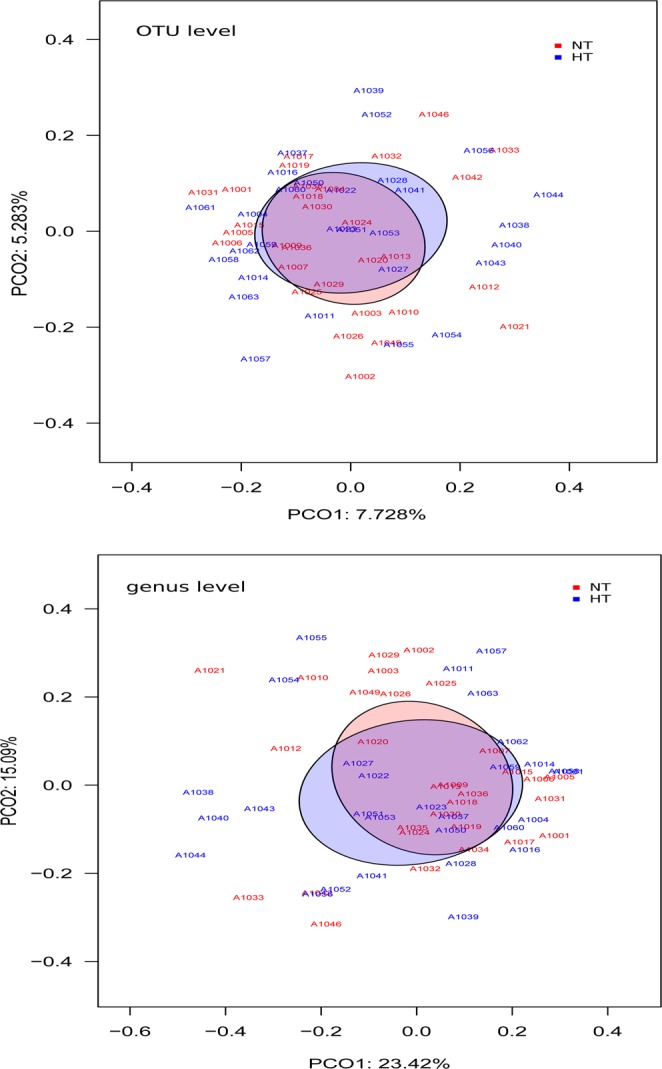
Beta diversity between NT and HT groups. PCoAs based on Bray-Curtis dissimilarity index at ASV and genus level.

To characterize the richness and diversity of bacterial community, we calculated, at ASV level, Chao1 estimator and Shannon index, respectively. As shown in Fig. 1b, the richness estimator and diversity index in the two groups were not significantly different. To assess the overall bacterial composition for both groups we performed Principal Coordinates Analysis (PCoA) using Bray-Curtis dissimilarity index at ASV and genus level (Fig. 2). No relevant differences in the gut microbiota at ASV (Adonis test, p = 0.31) or genus level (Adonis test, p = 0,33) were detected.

In contrast to our results, a previous study^23^ reported a significant dysbiotic gut microbiome in HT patients observing a decrease in the microbial richness and diversity compared to a healthy control group. This could be explained because in this previous study, a great part of HT patients had taken antihypertensive drugs and they were in a more advance hypertension grade. It has been recently reported that the hypertensive drug treatment can induce compositional changes in the gut microbiota and also inflammation^24^, so drug-induced gut microbiome shifts could have strongly influenced in their results. Moreover, in contrast to our study, in which we assessed diet composition and other lifestyle outcomes such as physical activity and sleep habits showing no significant differences among both groups, they did not control any environmental factor that could also affect the gut microbial community. In the present study, instead of observing a clear dysbiosis in HT, we report that subjects in a grade I of hypertension prior to drug treatment could have specific alterations in particular bacterial taxa.

**Figure 3:**
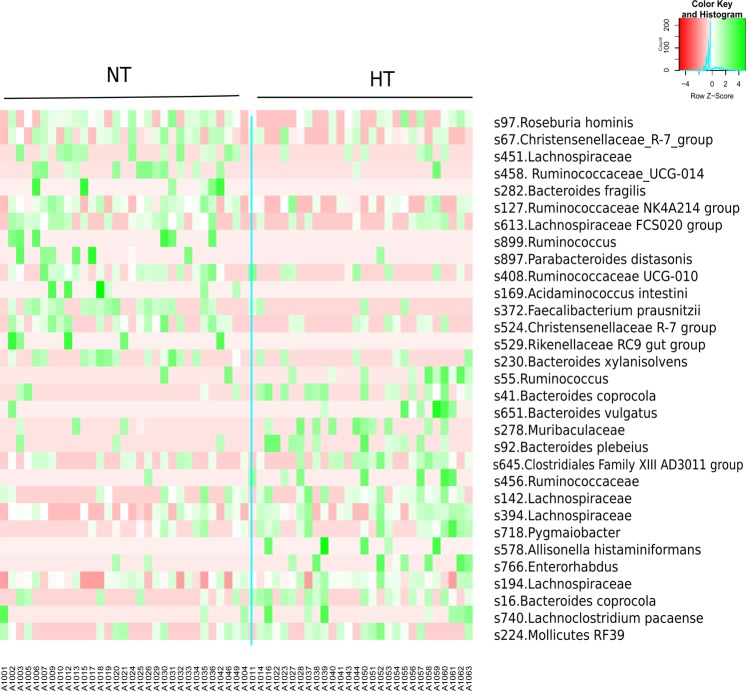
Heatmap based on ASV biomarkers. Only the ASVs having a LDA score >2.5 (log10) are represented. The relative abundance is expressed as Z score.

In order to identify specific taxa as biomarkers, we performed linear discriminant analysis (LDA) effect size (LEfSe) at ASV level. We detected, with LDA score > 2, a total of 67 ASVs biomarkers that had significant different abundance between HT and NT subjects (**Additional file 4:** Fig. S2). Figure 3 shows the normalized relative abundance (Z score) of the biomarkers that presented higher discriminant power (LDA score > 2.5) (Table 2). Particularly, we observed that in HT group three ASVs (s41, s16 and s92) with the highest LDA scores (3.08, 3.05 and 2.9, respectively) belong to *Bacteroides* genus. Two of them (s41 and s16) were classified as *Bacteroides coprocola* while the other ASV (s92) was assigned as *Bacteroides plebeius*.

**Table 2:** Significant differences between the two groups HT and NT in the relative abundance of ASVs that present a LDA score > 2.5 in LEfSe analysis.

ASV	LDA score (log10)	p.value	Taxonomy
Normotensive (NT)			
s897	2.5004	0.0267	p__Bacteroidetes;c__Bacteroidia;o__Bacteroidales;f__Tannerellaceae;g__Parabacteroides;s__distasonis
s529	2.5557	0.0229	p__Bacteroidetes;c__Bacteroidia;o__Bacteroidales;f__Rikenellaceae;g__Rikenellaceae_RC9_gut_group
s613	2.5759	0.0067	p__Firmicutes;c__Clostridia;o__Clostridiales;f__Lachnospiraceae;g__Lachnospiraceae_FCS020_group
s899	2.5799	0.0119	p__Firmicutes;c__Clostridia;o__Clostridiales;f__Ruminococcaceae;g__Ruminococcus
s408	2.6117	0.0146	p__Firmicutes;c__Clostridia;o__Clostridiales;f__Ruminococcaceae;g__Ruminococcaceae_UCG-010
s524	2.6530	0.0083	p__Firmicutes;c__Clostridia;o__Clostridiales;f__Christensenellaceae;g__Christensenellaceae_R-7_group
s169	2.6650	0.0119	p__Firmicutes;c__Negativicutes;o__Selenomonadales;f__Acidaminococcaceae;g__Acidaminococcus;s__intestini
s451	2.7061	0.0435	p__Firmicutes;c__Clostridia;o__Clostridiales;f__Lachnospiraceae
s282	2.7181	0.0119	p__Bacteroidetes;c__Bacteroidia;o__Bacteroidales;f__Bacteroidaceae;g__Bacteroides;s__fragilis
s458	2.7226	0.0042	p__Firmicutes;c__Clostridia;o__Clostridiales;f__Ruminococcaceae;g__Ruminococcaceae_UCG-014
s97	2.7644	0.0199	p__Firmicutes;c__Clostridia;o__Clostridiales;f__Lachnospiraceae;g__Roseburia;s__hominis
s230	2.7885	0.0339	p__Bacteroidetes;c__Bacteroidia;o__Bacteroidales;f__Bacteroidaceae;g__Bacteroides;s__xylanisolvens
s67	2.8589	0.0174	p__Firmicutes;c__Clostridia;o__Clostridiales;f__Christensenellaceae;g__Christensenellaceae_R-7_group
s127	2.8661	0.0060	p__Firmicutes;c__Clostridia;o__Clostridiales;f__Ruminococcaceae;g__Ruminococcaceae_NK4A214_group
s372	2.91	0.0005	p__Firmicutes;c__Clostridia;o__Clostridiales;f__Ruminococcaceae;g__Faecalibacterium;s__prausnitzii
Hypertensive (HT)			
s651	2.5177	0.0219	p__Bacteroidetes;c__Bacteroidia;o__Bacteroidales;f__Bacteroidaceae;g__Bacteroides;s__vulgatus
s740	2.5255	0.0368	p__Firmicutes;c__Clostridia;o__Clostridiales;f__Lachnospiraceae;g__Lachnoclostridium;s__pacaense
s766	2.5713	0.0260	p__Actinobacteria;c__Coriobacteriia;o__Coriobacteriales;f__Eggerthellaceae;g__Enterorhabdus
s645	2.5868	0.0494	p__Firmicutes;c__Clostridia;o__Clostridiales;f__Family_XIII;g__Family_XIII_AD3011_group
s456	2.6274	0.0191	p__Firmicutes;c__Clostridia;o__Clostridiales;f__Ruminococcaceae
s718	2.6301	0.0244	p__Firmicutes;c__Clostridia;o__Clostridiales;f__Ruminococcaceae;g__Pygmaiobacter
s578	2.6434	0.0186	p__Firmicutes;c__Negativicutes;o__Selenomonadales;f__Veillonellaceae;g__Allisonella;s__histaminiformans
s394	2.6554	0.0238	p__Firmicutes;c__Clostridia;o__Clostridiales;f__Lachnospiraceae
s55	2.6743	0.0132	p__Firmicutes;c__Clostridia;o__Clostridiales;f__Ruminococcaceae;g__Ruminococcus
s194	2.7215	0.0434	p__Firmicutes;c__Clostridia;o__Clostridiales;f__Lachnospiraceae
s224	2.7357	0.0488	p__Tenericutes;c__Mollicutes;o__Mollicutes_RF39
s278	2.8638	0.0345	p__Bacteroidetes;c__Bacteroidia;o__Bacteroidales;f__Muribaculaceae
s142	2.8795	0.0147	p__Firmicutes;c__Clostridia;o__Clostridiales;f__Lachnospiraceae
s92	2.8831	0.0104	p__Bacteroidetes;c__Bacteroidia;o__Bacteroidales;f__Bacteroidaceae;g__Bacteroides;s__plebeius
s16	3.0552	0.0050	p__Bacteroidetes;c__Bacteroidia;o__Bacteroidales;f__Bacteroidaceae;g__Bacteroides;s__coprocola
s41	3.0803	0.0017	p__Bacteroidetes;c__Bacteroidia;o__Bacteroidales;f__Bacteroidaceae;g__Bacteroides;s__coprocola.

On the other hand, different genera of Ruminococcaceae, Lachnospiraceae, Acidaminococcaceae and Christensenellaceae families from Firmicutes phylum and Tannerellaceae, Rikenellaceae and Bacteroidaceae from Bacteroidetes, were the most frequent taxa in NT group. *Faecalibacterium prausnitzii* (LDA score = 2.91, p = 0.0005) presented the highest discriminant power in the NT group. This specie and *Roseburia hominis* (LDA score = 2.76, p = 0.02), both biomarkers for NT, have been described as the main SCFA-producers in healthy status^15,25^. Several species of the Ruminococcaceae genera were also enriched in the NT group (s127, s458, s408, s899). Ruminococcaceae genera can degrade several types of polysaccharides in the lower GI tract, including starch, cellulose, and xylan^26^. This genera also consume hydrogen and produce acetate that can be utilized by *Roseburia* to produce butyrate^27^. In accordance with previous human cohort studies in which gut microbiome in hypertension status was compared to healthy subjects^10,23^, a reduction in all of these bacterial groups in HT patients was also observed. The depletion of these specific bacteria may have functional consequences on SCFA production and therefore the ability of the host to repair the epithelium and to regulate inflammation.

### Correlation of biomarkers with clinical variables

We explored the correlations between microbiota composition, by means of ASV biomarkers, and clinical variables taking into account the confounders of age, waist circumference, LDL-cholesterol, BMI, fat mass and dietary fiber intake (Table 3). After adjusting by false discovery rate a few correlations remained significant.

**Table 3:** Correlations between ASV biomarkers and clinical variables.

ASV increased in HT group	Clinical variable	Spearman correlation index	p.value Spearman correlation	p.adjust Spearman correlation
s718. Ruminococcaceae, *Pygmaiobacter*	SBP	0.4586	0.0003	0.0399
s718. Ruminococcaceae, *Pygmaiobacter*	DBP	0.3565	0.0056^a^	0.1238
s394. Lachnospiraceae	FBG	0.4208	0.0009	0.0492
s394. Lachnospiraceae	SBP	0.3966	0.0019	0.0755
s394. Lachnospiraceae	DBP	0.3242	0.0122	0.1542
s394. Lachnospiraceae	Fat mass	0.3561	0.0076	0.1283
s1498. Ruminococcaceae	FBG	0.2894	0.0262^b^	0.2304
**ASV increased in NT group**	**Clinical variable**	**Spearman correlation index**	**p.value Spearman correlation**	**p.adjust Spearman correlation**
s230. *Bacteroides xylanisolvens*	cLDL	−0.3318	0.0103	0.1416
s451. Lachnospiraceae	TG	−0.3346	0.0096	0.1359
s372. *Faecalibacterium prausnitzii*	DBP	−0.4334	0.0006	0.0417
s372. *Faecalibacterium prausnitzii*	SBP	−0.4031	0.0015	0.0750
s524. Christensenellaceae_R-7_group	DBP	−0.3054	0.0186^c^	0.1871
s524. Christensenellaceae_R-7_group	SBP	−0.3075	0.0178^d^	0.1830
S458. Ruminococcaceae_UCG-014	DBP	−0.3402	0.0083^e^	0.1283

p is probability at α = 0.05.

SBP, systolic blood pressure; DBP, diastolic blood pressure; TG, tryglicerides; cLDL low density liproprotein cholesterol; FBG, fasting blood glucose. Variables were adjusted for age, waist circumference, cLDL, BMI, fat mass and total fiber intake.

^a^Confounding factor: Fat mass (p value = 0.058).

^b^Confounding factor: Fat mass (p value = 0.0614).

^c^Confounding factors: Age (p value = 0.0514), waist circumference (p value = 0.1676); total fiber intake (p value = 0.0578).

^d^Confounding factors: Waist circumference (p value = 0.092); total fiber intake (p value = 0.054).

^e^Confounding factor: Fat mass (p value = 0.067).

Two ASV biomarkers of HT group, s394 and s718, correlated positively with SBP (*r* 0.396, p = 0.0018 and p-adjust = 0.007, and *r* 0.46, p = 0.00026 and p-adjust = 0.039, respectively). These results indicated that higher relative abundance of these ASVs, implied higher values of SBP, suggesting a negative effect of these bacteria on health status. ASV s718 was assigned as *Pygmaiobacter*, a new acidogenic genus of Ruminococcaceae family^28^, was positively correlated with both SBP and DBP, and also appeared to be a biomarker of HT group (Table 2). ASV s394 was classified as a genus of Lachnospiraceae family and we found that its 16S rRNA sequence share by BLAST 97.27% of identity with *Faecalicatena orotica*, a new specie described by Sakamoto, M. *et al*.^29^. This fecal bacteria also displayed a positive correlation with DBP (*r* 0,324; p = 0,012 and p-adjust = 0.1542), fat mass (*r* 0.356, p = 0.007 and p-adjust = 0.1283) and fasting blood glucose (*r* 0.42, p = 0.0009 and p-adjust = 0.0492), so further studies would be needed to confirm the contribution of these bacteria in hypertension.

We reported that *Faecalibacterium prausnitzii (*ASV s372), biomarker of NT group with the highest discriminant power, was negatively correlated with SBP (*r* −0.403, p = 0.0015 and p-adjust = 0.075) and DBP (*r* −0.43, p = 0.0006 and p-adjust = 0.042) (Table 3). *F. prausnitzii* is a well-known butyrate-producer in the gut and this SCFA has a potent anti-inflammatory effect that could affect to BP^30^. Another NT biomarker (ASV s524) that was assigned as Christensenellaceae R-7 group also correlated negatively with SBP (r -0.308, p = 0.018 and p-adjust = 0.18) and DBP (*r* −0.305, p = 0.0186 and p-adjust = 0.187). Christensenellaceae family has been described also as SCFA producer and associated with low BMI and with reduced weight gain in mice^31^.

### Identification of hypertension-associated markers from gut microbiome

**Figure 4:**
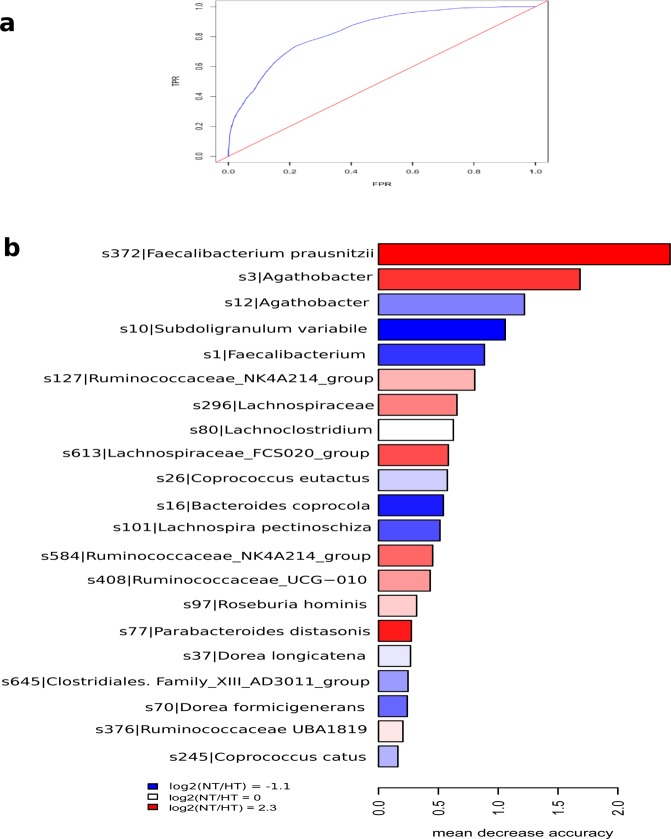
Random forest analysis at ASV level. **(a)** ROC curve using ASVs as variables. FPR, False Positive Rate, TPR, True Positive Rate. **(b)** The most discriminatory ASVs to classify individuals into NT or HT group. The colour indicates the enrichment in NT (red) or HT (blue) based on the log2 fold change between the relative abundance average of NT group and the relative abundance average of HT group. Length of bars represents the discriminatory power of the variables.

To determine the discriminant power of microbiota signatures in the identification of hypertension state, we applied random forest analysis. We obtained the discriminatory power of the area under the ROC curve (AUC) of 0,84 (Fig. 4a). 21 ASVs allowed to discriminate between HT patients and healthy subjects (p = 0,0017, Adonis test).

In line with LEfSe results, *B. coprocola* (HT-enriched) and *F. prausnitzii, Roseburia hominis*, Ruminococcaceae UCG-010, Lachnospiraceae FCS020_group and Ruminococcaceae NK4A214 group (HT-depleted), featured as microbiota members for the discrimination between groups (Fig. 4b). The results suggested that these specific bacterial taxa could have a strong relation with hypertension, however, more studies are needed to elucidate the mechanisms involved in this pathology.

### Differences in microbiota metabolic functions

To explore functional hallmarks, we performed a metagenomic analysis in both groups. The sequencing of the metagenomes yielded a total of 4.76 Gb with an average of 78 Mb per sample. The functional composition of the microbiota was elucidated comparing all the ORFs with TIGRFAM database of prokaryotic protein family models, obtaining an assignment of 35,6% of the ORFs (82,269 genes per sample). TIGRFAM protein family model is a hierarchical classification entailing main roles, the highest functional levels, and subroles, which represent more specific functions within each main role. PCoA, based on Bray-Curtis dissimilarity index, indicated a similar functional composition at TIGRFAM protein family (TIGRFAM) and subrole level (**Additional file 5:** Fig. S3). In accordance with previous work^10,23^, we detected, using LEfSe package, that NT group presented higher abundance of the subrole “Signal Transduction_Two Component Systems” (p = 0.015) (**Additional file 6:** Fig. S4). On the other hand, HT-associated microbiota was enriched in genes involved in the energy metabolism (subroles: Electron Transport, p = 0.04 and Anaerobic, p = 0.045), cellular processes (subrole DNA transformation, p = 0.03) and DNA metabolism (subrole DNA replication, recombination and repair, p = 0.042). Although the differential relative abundance in SCFA-producer bacteria reported, no significant differences have been detected in SCFA-producing pathways between NT and HT groups.

**Figure 5:**
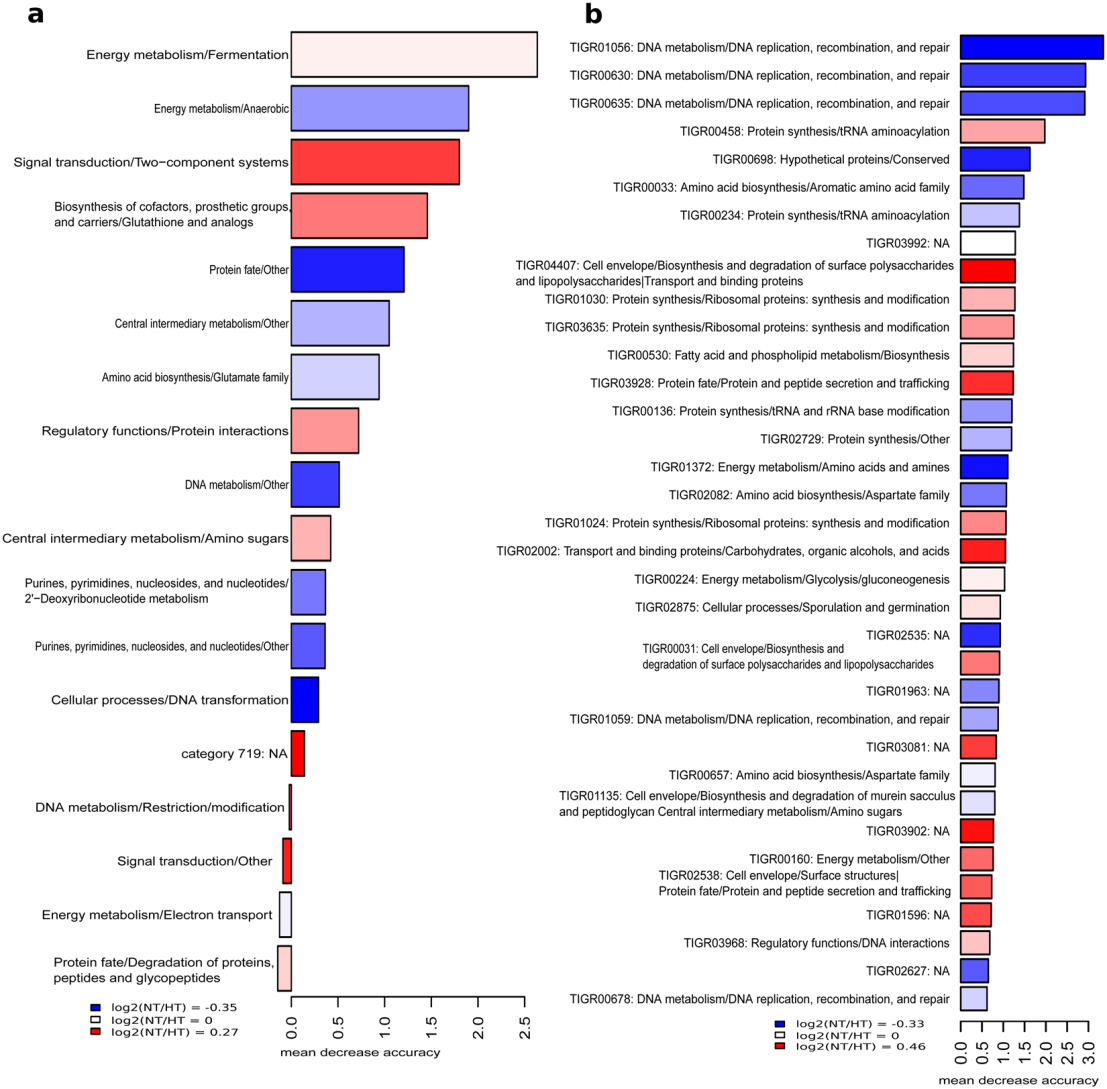
Random forest using **(a)** functional subroles and **(b)** TIGRFAM as discriminatory variables. The colour indicates the enrichment in NT (red) or HT (blue) based on the log2 fold change between the relative abundance average of NT group and the relative abundance average of HT group. Length of bars represents the discriminatory power of the variables.

We assessed functional discrimination of hypertension status applying random forest model with subroles and TIGRFAM. The discriminatory model achieved an average AUC of 0.66 based on 18 functional subroles (Fig. 5a). However, the variable TIGRFAM was more powerful to distinguish NT and HT groups with an average AUC of 0.86 (Fig. 5b). We found that three TIGRFAMs involved in the pathway “DNA replication, recombination and repair” were more abundant in HT group and presented the highest discriminatory capacity. In contrast with Li *et al*. (2017)^10^, the discriminatory TIGRFAMs, belonging to the functional categories related to secretion and transport systems as well as biosynthesis of lipopolysaccharides and transport were decreased in HT patients (Fig. 5b).

### SCFA in feces and plasma

**Figure 6:**
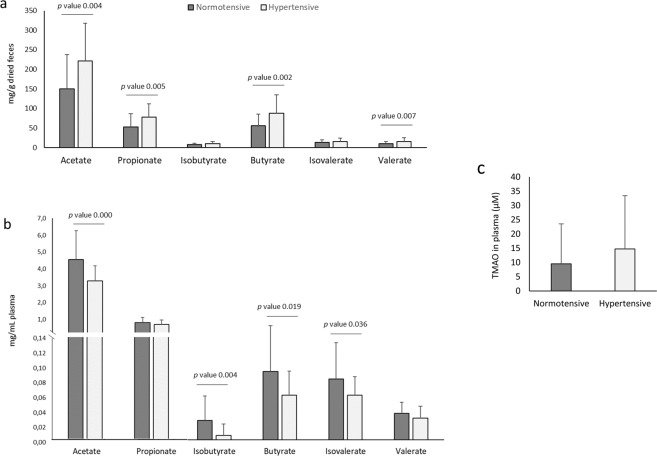
Mean values ± standard deviation of bacterial metabolites detected in plasma and feces. **(a)** Short chain fatty acids in feces expressed as mg/g dried feces (n = 61), (**b)** short chain fatty acids in plasma expressed as mg/mL (n = 61) and (**c)** trimethylamine N-oxide in plasma expressed as μmol/L (n = 59).

Recent studies have found that changes in BP often coordinate with changes in SCFA^32^. So in the present study, SCFA were reported separately in feces and plasma of HT and NT subjects (Fig. 6). In feces, HT showed significantly higher concentrations of acetate (p = 0.004), propionate (p = 0.005), butyrate (p = 0.002) and valerate (p = 0.003) compared to NT subjects (Fig. 6a). Similarly to our findings, some animal studies found elevated levels of fecal acetate and propionate in HT rat model in contrast to control rats^33,34^. Another study conducted in rats, observed significant elevated levels of acetate in salt sensitive rats which received a microbial transplant that increased their BP^9^. In line with these results, de la Cuesta-Zuluaga *et al*. (2018)^15^ examined in 441 subjects associations of fecal SCFA, gut microbiota and cardiometabolic outcomes, and they observed that higher fecal SCFA concentrations were associated with a measure of gut permeability and hypertension.

Previous cross-sectional studies have also reported higher fecal SCFA concentrations in overweight or obese individuals compared to lean individuals^35–38^. Our study corroborates these results, reporting also a significant positive correlation of SBP with fecal acetate (*r* 0,37; p = 0,003), propionate (*r* 0,35; p = 0,005) and butyrate (*r* 0,36; p = 0,004) (**Additional file 7:** Table S3).

SCFA are rapidly and efficiently absorbed in the colon with less than 5% being excreted in feces. Fecal SCFA concentrations have been used to determine SCFA production, however they are also a surrogate measure of SCFA absorption in the colon. Indeed, it has been suggested that fecal SCFA concentration could better represent SCFA absorption than its production^39^. So in the present study, we also analysed these fermentation products in plasma to study the SCFA absorption grade. To the best of our knowledge, no previous human studies have assessed both fecal and circulating SCFA in HT patients. Remarkably, plasma results showed an opposite trend compared to fecal results, reporting in HT group significant lower levels of circulating acetate (p = 0.000), isobutyrate (p = 0.004), butyrate (p = 0.019) and isovalerate (p = 0.036) compared to NT (Fig. 6b). A recent study performed in SHR, showed the same antagonistic results, reporting higher fecal butyrate levels in contrast to circulation levels^40^. They also reported lower expression levels of butyrate‐sensing receptors in the hypothalamus of SHR and reduced expression of Slc5a8 transporter in the colon, so they concluded that a reduced availability of serum butyrate in the SHR is possibly due to a diminished absorption in the colon. Our study could corroborate this hypothesis in humans, and also extends these results, not only to butyrate, but also to other SCFA, such as acetate, the most abundant SCFA in plasma (Fig. 6).

Other recent studies may reinforce this hypothesis, reporting a strong relation between hypertension, gut mucosal permeability and altered inflammatory status in rats^11^ and humans^41^ with hypertension. Kim *et al*. reported significant increases in plasma of intestinal fatty acid binding protein, lipopolysaccharide, and augmented gut-targeting proinflammatory T helper 17 cells in high BP patients, which demonstrated increased intestinal inflammation and permeability in HT subjects^41^. Together with these results, they also observed significant lower levels of plasma butyrate in HT subjects.

Since our study demonstrates that HT subjects present significant depleted levels of butyrate-producing bacteria and plasma SCFA together with higher levels of SCFA in feces, our results strengthen the hypothesis that a lower efficiency in the absorption of SCFA could occur in HT subjects and that an imbalanced host-microbiome crosstalk may be an important cause of hypertension. Moreover, no significant differences were observed in bacterial pathways related to SCFA biosynthesis, which reinforces this hypothesis.

Another way that SCFA can influence host cells, especially acetate and propionate, is by cellular mechanisms related to host GPCRs involved in SCFA signalling^32^. Intriguingly, Gpr41 and Olfr78 play opposite roles in BP regulation. While Gpr41 null mice are hypertensive, the Olfr78 null mice are hypotensive^13,42^, which points out the physiological importance of these SCFA signalling pathways on the BP control. Many studies support the hypothesis that SCFA induce vasorelaxation^43,44^, which may be due to an increase in vascular tone regulated by the action of SCFA on Gpr41 in the vascular endothelium. Our results provide human evidence that significant lower levels in circulation of SCFA, especially acetate, the most abundant SCFA in plasma, could be in part a causative factor for the higher BP levels in HT subjects.

### Correlation of SCFA with clinical variables and diet

The relation between microbial metabolites, clinical variables and dietary parameters was also tested (**Additional file 7:** Table S3). As indicated, most of fecal SCFA detected in feces appeared to have a positive correlation with SBP and DBP. When we tested the relationships between plasma SCFA and BP, we only found a negative correlation between DBP and acetate (*r* = −0,26, p = 0.045), isobutyric (*r* = −0,33, p = 0.009) and isovaleric acid (*r* = −0,30, p = 0.017), and between SBP and isobutyric acid (*r* = −0,42, p = 0.001). However, other positive correlations were observed between anthropometrical parameters such as weight, BMI and waist circumference and fecal acetate, propionate, butyrate, valeric acid, isobutyrate and isovaleric acid, indicating that other variables could have influenced in the SCFA production or absorption efficiency.

In relation to diet, clinical studies have shown that a high intake of fruit and vegetables, considered sources of SCFA, is associated with reduced BP levels^45^. In the present study, the intake of fruits and vegetables was not different between both groups but the NT group presented a higher total dietary fiber intake, probably due to other foods rich in fiber such as whole-grain cereals **(Additional File 3:** Table S2). Despite that, a negative correlation was observed between fecal propionate, valeric and isobutyric acids and total dietary fiber intake (**Additional file 7:** Table S3), which could be related to the lower levels of fecal SCFA detected in NT compared to HT subjects. HT group also presented a higher intake of starch food sources such as potatoes, and starches presented a positive correlation with plasma propionate. These results could be related to the fermentation of resistant starches that contribute positively to colonic SCFA production as previously reported^46^.

### TMAO in plasma

No significant differences in fasting TMAO concentrations between HT and NT groups were found (Fig. 6c). Although a large body of evidence supports that TMAO is involved in atherosclerosis, the existing body of literature is sparse regarding TMAO and hypertension. Only in HT rats an increased permeability of the colonic gut-blood barrier to TMA, the main TMAO precursor, has been evidenced^47^, but the role of TMAO in HT humans remains unclear.

Despite not reporting significant differences between groups, results from Pearson correlations showed significant positive relationships between TMAO and total cholesterol (*r* = 0.28, p = 0.030), LDL-cholesterol (*r* = 0.30, p = 0.019) and fasting blood glucose (*r* = 0.27, p = 0.034) (**Additional file 7:** Table S3). In fact, TMAO molecule has been identified as a novel biomarker of cardiovascular risk, and increased levels have been negatively associated with ‘reverse cholesterol transport’ and also with defects in cholesterol metabolic pathways^48,49^. Similarly to our findings, a cross-sectional study performed with subjects at risk for type 2 diabetes found positive relationships of fasting TMAO concentrations with total- and LDL-cholesterol, and fasting glucose^50^.

As observed (Fig. 6c), a great inter-individual variability was reported in fasting TMAO concentration. In accordance with a recent review, circulating TMAO levels are influenced by several factors including gut microbiota composition and activity, liver function and excretion, gut-blood barrier function and diet^51^. In the present study, we found no significant associations between TMAO and dietary food sources reported by 3-day dietary records. This may be explained in part by the short time elapsed between the intake of TMAO-rich food (such as fish) or its dietary precursors (choline and carnitine found in meat and eggs), and the appearance of the metabolite in plasma, as reported by Cho *et al*.^52^. As suggested by other authors^53^, high inter-variation in TMAO plasma levels, may be attributed to differences in gut microbiota composition and function. Some families of bacteria from Firmicutes and Proteobacteria phyla are potent choline and carnitine consumers, and are able to synthetize TMA through the expression of specific enzymes. In addition, other biochemical factors such as hepatic FMO3 expression and activity can affect TMAO levels. Hence, the highly variable plasma levels in a disease versus non-disease state are influenced by differences in gut bacterial composition, and it does not necessarily have to be a marker in the disease process. Therefore, for future studies, further assessment of TMAO status is needed and it should include the collection of repeated samples at different times and the determination of the average levels.

## Conclusions

The present study revealed that individuals with hypertension in a first disease grade prior to drug treatment possess a particular fecal bacterial signature. Our results provide a new approach to distinguish HT individuals from healthy subjects according to a specific bacterial and SCFA profile, despite the non-significant results in the overall composition of bacterial community. Specifically, HT subjects were characterized by lower abundance of SCFA producers *Faecalibacterium prausnitzii, Roseburia hominis*, Ruminococcaceae NK4A214, Ruminococcaceae_UCG-010, Christensenellaceae_R-7, and higher abundance of *Bacteroides coprocola, Bacteroides plebeius* and genera of *Lachnospiraceae*. Some of these bacterial taxa appeared to be positively and negatively associated with SBP and DBP after adjustment for confounders such as fiber intake, age and anthropometric variables. These results indicate that these taxa-specific differences detected in HT are not explained by the potential confounders and, therefore, they could be intrinsically related to hypertension. We also corroborate that SCFA fecal levels do not reflect SCFA levels in circulation, highlighting the importance of analysing SCFA in plasma, where they can be sensed by host GPCRs. As far as we are aware, this is the first time to show that higher fecal excretion of most of the SCFA (acetate, propionate, butyrate, valerate) together with lower plasmatic levels (acetate, isobutyrate, butyrate, isovalerate) is associated with hypertension in humans. In accordance with previous animal studies, we hypothesize that these opposed results could indicate a less efficient SCFA absorption in HT subjects. Further in-depth research must be done to elucidate how differ the production and metabolism of SCFA between HT and NT and to better understand the potential connection between hypertension and SCFA.

Despite the relevant results presented here, some limitations of the study must be noted. The number of subjects was low and not strictly homogenous in terms of age and several clinical variables. Also, the grade I hypertension in HT subjects could be influenced by the white-coat and masked hypertension. To minimize this effect, we monitored SBP and DBP by using multiple automated sphygmomanometer in two repeated readings, and with participants resting in a sitting position alone and unobserved in a quiet environment. By applying these practices the white-coat effect can be substantially reduced or eliminated^1^.

Overall, our results present a disease classifier based on microbiota and bacterial metabolites to distinguish HT from NT individuals in a first disease grade prior to drug treatment. Moreover, it is newly reported that HT subjects showed a particular SCFA profile in feces and plasma that could indicate a less efficient SCFA absorption.

## Materials and Methods

### Subjects and study design

All individuals included in the present study were recruited between 9 June 2016 and 28 November 2017. Participants were recruited in Reus (Spain) by using tableaux advertisements in the Hospital Universitari Sant Joan (HUSJ), and using databases of volunteers who have previously participated in studies carried out in our research group. HT participants were included into the study if they were at grade I hypertension, defined as SBP between 140 and 159 mmHg and without major complications according to the ESC/ESH Guidelines (2018)^1^, and were not using antihypertensive treatment. NT participants were included if presented optimal SBP < 120 mmHg. Additionally, SBP between 140 and 159 mmHg also correspond to stage 1 hypertension defined by the US -JNC7 report^54^.

All participants fulfilled the following criteria: to be aged from 18 to 65, without family history for cardiovascular disease or evidence of chronic disease, and with willingness to provide informed consent before the initial screening visit. Subjects with BMI ≥ 30 kg/m^2^; fasting glucose > 126 mg/dL; LDL-cholesterol > 190 mg/dL; triglycerides > 350 mg/dL, smoking, and suffering anemia or intestinal disorders were excluded. Individuals were also excluded if they had received antibiotics or probiotics within the last 3 months, and if they were following a vegetarian diet.

A total of 61 participants, 29 HT and 32 NT, were enrolled in the study. Volunteers attended to 2 visits at the HUSJ and Eurecat-Reus where the study was performed. In a first pre-selection visit, a clinical interview to verify that participants met all the eligibility criteria was done.

Study was approved by local ethics committee (Clinical Research Ethical Committee of HUSJ, Reus with 15-11-26/11obs4 reference) and informed consent was obtained from all subjects. The protocol and trial were conducted in accordance to the Helsinki Declaration and Good Clinical Practice Guidelines of the International Conference of Harmonization (ICH GCP).

### Biological samples collection

Blood samples were collected at fasting state in serum and plasma blood collection tubes. Both samples were collected and kept at −80 °C until the further biochemical analysis.

Participants received detailed instructions to collect fecal samples. They were provided with a Protocult^TM^ stool collection device (ABC, Minnesota, EEUU) and two different containers: a sterile pot and a specimen container with spoon containing 10 mL of RNAlater^®^ storage solution (Sigma-Aldrich Quimica SL; Madrid, Spain). The volunteers were asked to transfer a small amount of fresh feces from the sterile pot to the other tube immediately after defecation and to freeze the samples. Volunteers transported the frozen samples to the laboratory with ice pack. Fecal samples from the sterile pot were lyophilized and stored at −80 °C until the chromatographic analysis to determinate SCFA. The feces preserved in RNAlater^®^, also stored at −80 °C, were used for the analysis of microbiota composition.

### Clinical outcomes measurement

All clinical information was collected according to standard procedures and was measured in both visits. SBP and DBP was measured in a sitting position after 2–5 minutes participants respite by using an automatic sphygmomanometer (OMRON HEM-907; Peroxfarma, Barcelona, Spain). Two readings were recorded with 1-min interval, and the average value was used for statistical analyses. Weight and body composition was measured by trained dietitians with a body composition analyzer (Tanita Leicester Portable; Tanita Corp., Barcelona, Spain). Waist circumference was measured at the umbilicus level using a 150 cm anthropometric steel measuring tape^55^.

### Lipid profile

A fasting blood sample was obtained in the second visit to determine lipid profile. Briefly, total cholesterol, LDL-cholesterol, high density lipoprotein cholesterol (HDL-cholesterol), triglycerides, Apolipoprotein A-1 (ApoA-1) and Apolipoprotein B-100 (ApoB-100) concentrations were measured in serum by standardized enzymatic automated methods in an autoanalyzer (Beckman Coulter-Synchron, Galway, Ireland).

### Lifestyle outcomes measurement

Diet composition was assessed through a 3-day dietary record (2 labor days and 1 week-end day) and calculated by Spanish Food Composition Tables^56^, and dietary habits were self-reported by volunteers through a validated, semi-quantitative, FFQ containing 137 food items related to the Mediterranean diet^57^. Physical activity was evaluated by completion of a validated questionnaire (Physical Activity Questionnaire Class AF)^58^. Usual sleep habits were assessed by Pittsburgh Sleep Quality Index (PSQI)^59^.

### Determination of fecal and plasma SCFA

For the sample extraction, 0.1 g of lyophilized feces were mixed with 1 mL of acidified aqueous solution (1% phosphoric acid) containing 4-methyl valeric acid (Sigma-Aldrich, St. Louis, MO, USA) as internal standard (IS, final concentration 500 μM). Samples were shaken for 15 min and centrifuged (10 min, 1800 × *g*, 4 °C). Before filtration (0.22 mm pore size filter), the supernatants were centrifuged (4 min, 8784 × *g*, 4 °C) once more. The analysis of acetic, propionic, butyric, isobutyric, isovaleric, and valeric acids was performed by gas chromatography (Agilent 7890 A Series) using a capillary BP-21 column (30 m, 0.25 mm, 0.25 μm; SGE, Cromlab SL, Barcelona, Spain), coupled to a flame ionization detector (GC-FID). The column temperature was programed at 90 °C, rising by 15 °C/min until it reached 150 °C, then 5 °C/min to 170 °C, and then 20 °C/min to 240 °C, and maintained 3 min (total run time 14.5 min). Helium was the carrier gas (1 mL/min). Injection was carried out with a split injector (1:100) at 220 °C, detector temperature was 250 °C, and 1 μL of the solution was injected into the GC-FID system. Identification of the SCFA was carried out according to the retention time of standard compounds (acetic acid, propionic acid, butyric acid, isobutyric acid, valeric acid, and isovaleric acid; Sigma-Aldrich) and their quantification was determined with reference to the peak side of IS (4-methyl valeric acid). All samples were analyzed in triplicate.

Quantification of SCFA in plasma was performed as described previously by Zhang *et al*. (2019)^60^, with some modifications. Briefly, for sample extraction 200 μL of plasma was mixed with 200 μL of milliQ water containing 2-methyl valeric as IS and with 200 μL of diethyl ether (DE) in a 0.6 mL microtube containing a drop of hydrochloric acid (37%). Samples were shaken for 5 min at 4 °C and centrifuged (15 min, 9000 rpm, 4 °C). The DE layer (containing SCFA) was transferred to a new 0.6 mL microtube containing a small amount of anhydrous Na_2_SO_4_ (to remove the residual water). The remaining aqueous layer was further extracted with DE for two more times. The DE layers were pooled and mixed for further derivatization. For the derivatization procedure 210 μl of DE extract was accurately transferred into a glass insert in a GC vial and capped tightly after added 20 μl of (trimethylsilyl)-trifluoroacetamide (BSTFA). The mixture was kept in the GC vial and incubated with agitation at 37 °C for 2 h, 37 °C. The analysis of SCFA was performed by gas chromatography (Agilent 6890N-MSD 5973) using a DB5 MS-UI column (30 m, 0.25 mm, 0.25 μm; J&W, Agilent Tech), coupled to a mass detector using SIM mode. 1 μl of derivatized sample was injected with split ratio of 1:5. All the procedure was performed at low temperature using ice and cooling the sample carousel. The contents of SCFA were calculated with internal standard method. All samples were analyzed in duplicate, reporting coefficient of variability values lower than 10%.

### TMAO in plasma

Quantification of TMAO in plasma samples was performed as described previously^61^. Briefly, for the sample extraction, 25 µL of plasma was mixed with 80 µL of methanol with labelled IS working solution (TMAO-d9; Cambridge Isotope Laboratories, Massachusetts, USA) and mixed 30 seconds to precipitate proteins. The samples were subjected to centrifugation at 9000 rpm for 5 min at room temperature, and the supernatants were diluted with 150 uL of MilliQ water. Diluted samples were filtered with PVDF filters 0.22 µm and transferred into HPLC vials for analysis. The analysis was performed by liquid chromatography coupled to tandem mass spectrometry (UPLC-MS/MS) (Waters, Milford, MA, USA) using a column Acquity UPLC BEH HILIC (1,7 µm 2,1 × 100 mm).

### Fecal microbiota analysis

#### DNA purification and sequencing

Fecal samples stored in RNAlater^®^ were diluted with PBS solution (dilution1:2). To remove fecal debris, the samples were centrifuged at 2000 rpm at 4 °C for 2 min. Total DNA was extracted from pelleted bacterial cells in the robotic workstation The MagNA Pure LC Instrument (Roche) using the MagNA Pure LC DNA isolation kit III (Bacteria, Fungi) (Roche) according to the manufacturer’s instructions. The region V3-V4 of the 16S rRNA gene was amplified by PCR and used to amplicon library construction following Illumina instructions. Metagenomic libraries were constructed using NEXTERA XT kit according to the manufacturer’s instructions (Illumina). Sequencing was performed with the Kit V3 (2 × 300 cycles) in MiSeq platform (Illumina, Eindhoven, Netherlands) in the Centre for Public Health Research (FISABIO-Salud Pública, Valencia, Spain). All sequences were deposited in the public European Nucleotide. Archive server under accession number PRJEB32411.

#### Sequence analysis

16S rRNA gene reads with low-quality score and short read length as well as potential chimeras were removed using DADA2 pipeline in R package^62^. We used DADA2 pipeline to create the ASV. The taxonomic information of the 16S rDNA sequences was obtained by comparison with SILVA database (v.132)^63^. We considered only annotations that were obtained with a bootstrap value greater than 0.8, leaving the assignation at the last well-identified level and consecutive levels as unclassified.

For metagenome analysis, sequence trimming, filtering by quality and removal of host sequences were performed using a custom pipeline. Overlapping paired-end reads were joined using FLASH-1.2.11^64^ applying default parameters. Next, we assembled the filtered sequences into contigs, using MEGAHIT v1.1.2^65^. To know the number of reads in each contig, the reads were mapped against the resulting contigs with bowtie2. Not assembled sequences were appended to the contigs. Subsequently, prediction of open reading frames (ORF) was implemented by the program Prodigal v2.6.3^66^. Functional assignment was carried out aligning the ORF dataset via HMMER (v3.1b2)^67^ against the TIGRFAM database of prokaryotic protein family models (v9.0)^68^. After obtaining the functional annotation and the alignment coordinates for each obtained match, these coordinates were used to identify putative genes within the contigs. Finally, we aligned the filtered sequencing reads to the putative annotated genes in the contigs via megaBLAST and we quantified the abundace of each gene by counting the aligned reads, using in-house R scripts.

### Statistical analysis and bioinformatics analysis

Statistical analysis of clinical parameters was performed using IBM SPSS version 23.0 (IBM SPSS, Inc, Chicago, IL, USA). The normality of variables was assessed by using the Kolmogorov-Smirnov test. The Mann-Whitney test was used for comparison of non-normally distributed variables. Student’s t-test was used for comparison of normally distributed variables. Fisher’s exact test was used for categorical variables comparisons.

Pearson correlation coefficients were calculated for relationships between bacterial metabolites, SCFA and TMAO, and clinical and dietary variables in both groups. Descriptive data were expressed as mean ± SD and percentages for categorical variables. The level of statistical significance was set at p < 0.05.

The alpha diversity (Chao1 richness estimator and the Shannon diversity index) was determined at ASV level using vegan library from the R package^62^. To analyze beta diversity, Bray-Curtis dissimilarity index was calculated on the base of the abundance matrix from the taxonomic and functional composition to quantify the compositional and functional dissimilarity between two different communities.

Box plots, Principal Coordinates analysis (PCoA), sample clustering Principal Component analysis (PCA) and heatmaps were generated with in-house R scripts. The pairwise comparisons of continuous variables were analyzed using Wilcoxon rank-sum test.

To statistically assess the effect of the environmental factors on the bacterial and functional composition, a multivariate analysis of variance based on dissimilarity tests (Adonis) was applied as implemented in the vegan library in R package (library “vegan” function “adonis”). To identify ASVs and metabolic pathways as biomarkers, we applied the linear discriminant analysis (LDA) effect size (LEfSe) algorithm^69^. We fixed an α-value <0.05 and the threshold used to consider a discriminative feature for the logarithmic LDA score was set at >2 or >2.5.

We assessed putative correlations between ASV biomarkers and clinical variables by means of a linear regression model setting ASV biomarkers as predictors and clinical variables as response variable. Moreover, we also computed a Spearman’s test for each association. Thus, only pairs with statistically significant association (p < 0.05), based on both criteria, were selected to further analysis. To address potential confounding correlations, we adjusted the previous linear regression model by adding one by one the confounding variables as regressors in order to guarantee that, even in the presence of these confounders, the variance explained by the clinical variables (or metabolites) was still statistically significant. We adjusted for age, waist circumference (cm), LDL-cholesterol (mg/dL), BMI (kg/m^2^), fat mass (%) and dietary fiber intake (g/day). All computations were performed using R base functionalities (R core package, 2014), in particular ‘lm’ and ‘cor.test’ functions.

To control the false discovery rate, we validated the statistical tests adjusting all p-values using the Benjamini-Hochberg correction^70^ in R package (library “stats”, function “p.adjust”).

### Random forest analysis

We applied random forest modeling^71^ to find discriminant taxa (biomarkers) capable of correctly assigning the health status of our individuals. This algorithm is very flexible at capturing complex interactions between biomarkers as occur in our data. Random forest begins analyzing a first model considering the whole set of features and provides, from this first step, a measure of importance associated to each taxon. Then, we analyze successively additional models including lower and lower taxa with higher and higher associated importance as long as the prediction performance of the models increases. Since the number of parameters of those successive models decrease, we also get lower and lower overfitting. To compute the prediction performance of the model at each step, we perform 100-fold cross-validation at equal rates for training and test to obtain false positive and true positive rates. Then, by varying the discrimination threshold for classification, we get a ROC curve for the model and score it by means of the Area Under this Curve (AUC), so higher AUC better prediction performance.

## Supplementary information

Supplementary information.
