# Supplementary material for: Gut metagenomic and short chain fatty acids signature in hypertension: a cross-sectional study
Source: Sci Rep. 2020 Apr 15;10:6436. doi: 10.1038/s41598-020-63475-w (PMC7160119; doi:10.1038/s41598-020-63475-w)

Gut metagenomic and short chain fatty acids signature in hypertension: a cross-sectional study

Lorena Calderón-Pérez[1,2,+], Maria José Gosalbes[3,4,+], Silvia Yuste[5], Rosa M Valls[2,1]*, Anna Pedret[1,2], Elisabet Llauradó[2], Nuria Jimenez-Hernandez[3,4], Alejandro Artacho[3,4], Laura Pla-Pagà[1,2], Judit Companys[1,2], Iziar Ludwig[1], Maria-Paz Romero[5], Laura Rubió[5]*, Rosa Solà[1,2,6]

[1] Eurecat, Centre Tecnològic de Catalunya, Unitat de Nutrició i Salut, Reus, Spain.

[2] Universitat Rovira i Virgili, Facultat de Medicina i Ciències de la Salut, Functional Nutrition, Oxidation, and Cardiovascular Diseases Group (NFOC-Salut), C/Sant Llorenç 21, 43201-Reus, Spain.

[3] Fundación para el Fomento de la Investigación Sanitaria y Biomédica, Valencia, Spain

[4] CIBER en Epidemiología y Salud Pública (CIBEResp) Madrid, Spain

[5] Food Technology Department, XaRTA-TPV, Agrotecnio Center, Escola Tècnica Superior d'Enginyeria Agrària, University of Lleida. Avda/Alcalde Rovira Roure 191, 25198-Lleida, Catalonia, Spain

[6] Hospital Universitari Sant Joan de Reus, Reus, Spain

+ LC and MJG contributed equally to the study

*Correspondence: laura.rubio@udl.cat; rosamaria.valls@urv.cat

Additional file 1. Figure S1. Flow diagram participants of study

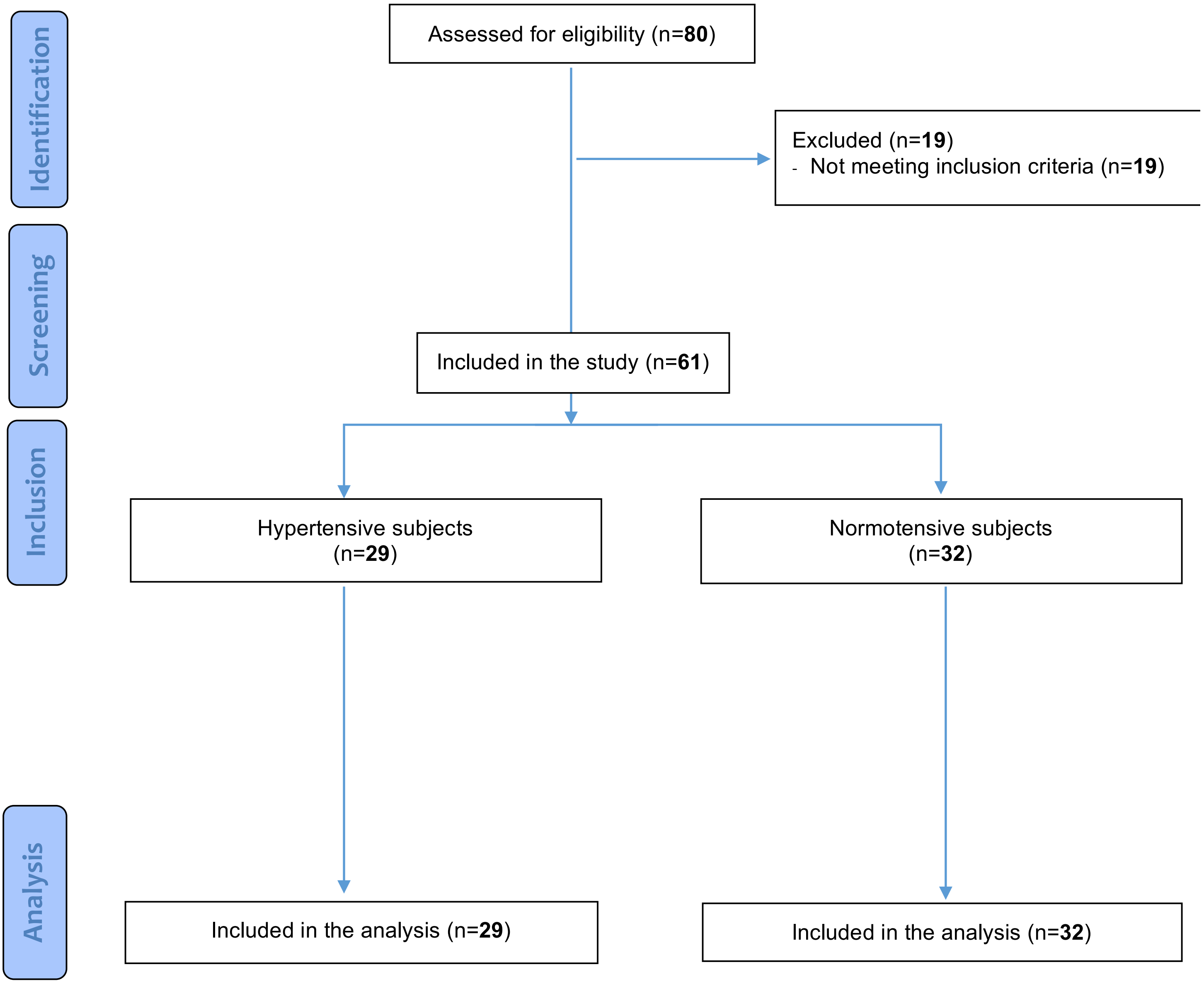

Additional file 2: Table S1 | Mean energy and daily nutrients intake.

**Nutrient**	**Hypertensive (n=28)**	**Normotensive (n=32)**	**P-value**
Energy (Kcal)	2089.74 ± 543.43	2208.23 ± 654.06	0.452
Protein *(g)*	88.28 ± 21.28	94.10 ± 29.87	0.395
Total carbohydrates *(g)*	197.03 ± 61.41	212.65 ± 60.90	0.328
Complex carbohydrates	110.35 ± 42.29	116.00 ± 27.31	0.548
Added sugars	87.87 ± 29.64	96.62 ± 40.38	0.767
Total fat *(g)*	96.95 ± 31.67	102.70 ± 41.93	0.556
SFA	27.54 ± 10.54	27.75 ± 12.47	0.944
MUFA	45.40 ± 15.63	49.67 ± 21.92	0.395
PUFA	15.92 ± 7.29	17.22 ± 8.58	0.563
Total cholesterol *(mg)*	328.25 ± 130.77	358.22 ± 187.89	0.482
Dietary fiber *(g)*	20.95 ± 9.90	25.90 ± 11.63	**0.029**
Ethanol *(g)*	11.37 ± 13.04	7.76 ± 10.11	0.131
Sodium *(mg)*	2371.75 ± 858.61	2433.50 ± 824.65	0.778
Potassium *(mg)*	3409.72 ± 718.93	3619.49 ± 1110.46	0.383

Data expressed as mean ± standard deviation. Daily mean nutrients intake was estimated from 3-day dietary record. SFA, saturated fatty acids; MUFA, monounsaturated fatty acid; PUFA, polyunsaturated fatty acid. P-value estimated by Student's t-test and Mann-Whitney U test. Significant differences depicted in **bold.**

Additional file 3: Table S2 | Mean intake in g/day by food group.

**Food group intake *(g/day)***	**Hypertensive (n=27)**	**Normotensive (n=32)**	**P-value**
Dairy products	240.1 ± 150.4	329.3 ± 295.3	0.513
Whole dairy	65.62 ± 59.35	103.4 ± 154.1	0.610
Semi-skimmed dairy	86.28 ± 112.3	105.5 ± 151.7	0.766
Skimmed dairy	76.28 ± 115.2	85.78 ± 112.7	0.926
Eggs	25.25 ± 12.80	27.11 ± 28.71	0.537
Meats	193.1 ± 122.2	148.6 ± 106.3	0.140
Red meat	64.95 ± 44.70	53.30 ± 50.40	0.150
White meat	82.09 ± 81.11	65.71 ± 78.23	0.125
Processed meat	46.03 ± 28.52	29.54 ± 25.13	**0.016**
Fish and seafood	103.9 ± 57.60	107.8 ± 71.10	0.976
Whitefish	37.40 ± 26.50	36.46 ± 27.75	0.691
Bluefish	42.86 ± 23.09	48.13 ± 43.72	0.517
Seafood	23.72 ± 18.93	23.20 ± 16.14	0.770
Vegetables	467.1 ± 261.4	433.0 ± 217.7	0.533
Potatoes	66.22 ± 38.16	40.37 ± 32.58	**0.005**
Fresh fruit	399.9 ± 239.9	403.7 ± 338.5	0.420
Nuts	38.20 ± 36.09	42.46 ± 45.45	0.802
Legumes	27.72 ± 8.78	25.45 ± 11.92	0.417
Cereals	213.5 ± 176.8	238.9 ± 188.4	0.553
Refined cereals	121.4 ± 86.69	104.1 ± 85.13	0.312
Whole-grain cereals	26.51 ± 42.88	50.63 ± 68.66	**0.034**
Oils and fats	23.13 ± 14.96	27.70 ± 21.99	0.503
Olive oil	20.50 ± 14.61	25.30 ± 22.18	0.374
Sunflower oil	0.36 ± 0.89	1.17 ± 2.64	0.511
Butter	2.03 ± 4.58	1.21 ± 1.73	0.407

Pastries	14.52 ± 12.60	20.39 ± 19.76	0.583
Chocolate	7.03 ± 7.95	6.36 ± 8.40	0.590
Pre-cooked food	53.70 ± 40.00	39.53 ± 23.82	0.191
Non-alcoholic beverages	235.8 ± 172.1	144.8 ± 136.2	**0.004**
Sugary carbonated drinks	28.85 ± 45.34	9.46 ± 16.31	0.190
Soft drinks	12.66 ± 34.15	22.70 ± 56.08	0.713
Commercial juices	30.63 ± 96.65	18.83 ± 50.27	0.574
Natural juices	85.32 ± 81.47	27.88 ± 45.24	**0.008**
Coffee	60.11 ± 45.30	44.70 ± 57.45	**0.033**
Tea	18.23 ± 33.01	21.18 ± 36.97	0.906
Alcoholic beverages	160.8 ± 145.2	131.7 ± 162.2	0.108
Wine	57.60 ± 81.08	41.35 ± 61.76	0.278
Beer	101.9 ± 102.9	88.83 ± 112.1	0.213
Distilled spirits	1.28 ± 2.56	1.52 ± 4.05	0.756

Data expressed as mean ± standard deviation. Daily mean intake by food groups was estimated from adapted Food Frequency Questionnaires performed in 59 subjects. P-value estimated by Student's t-test and Mann-Whitney U test. Significant differences depicted in **bold.**

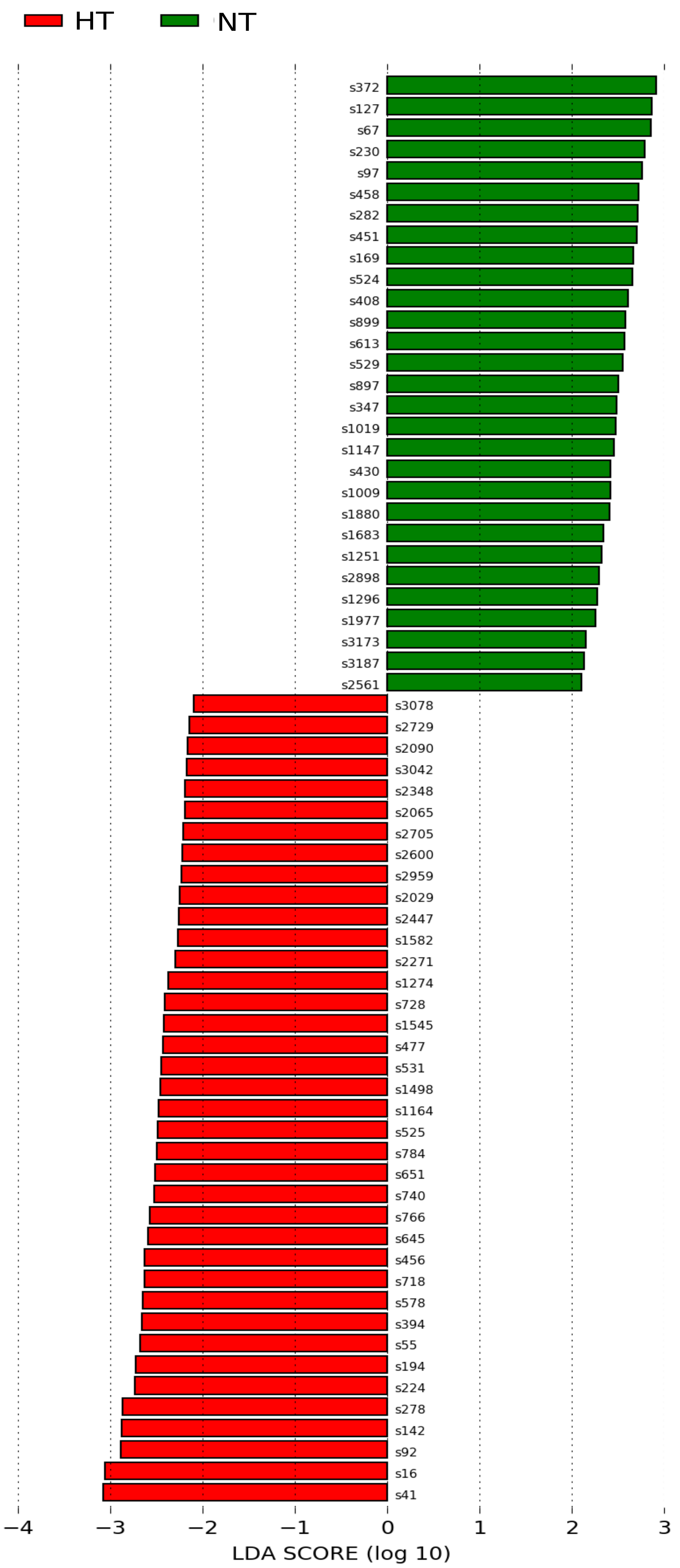

Additional file 4: Figure S2: LefSe analysis of ASVs between NT (green) and HT (red). LDA scores (log10) for the most prevalent ASV in NT group are represented in positive scale whereas LDA-negative scores indicated enriched ASV in HT group.

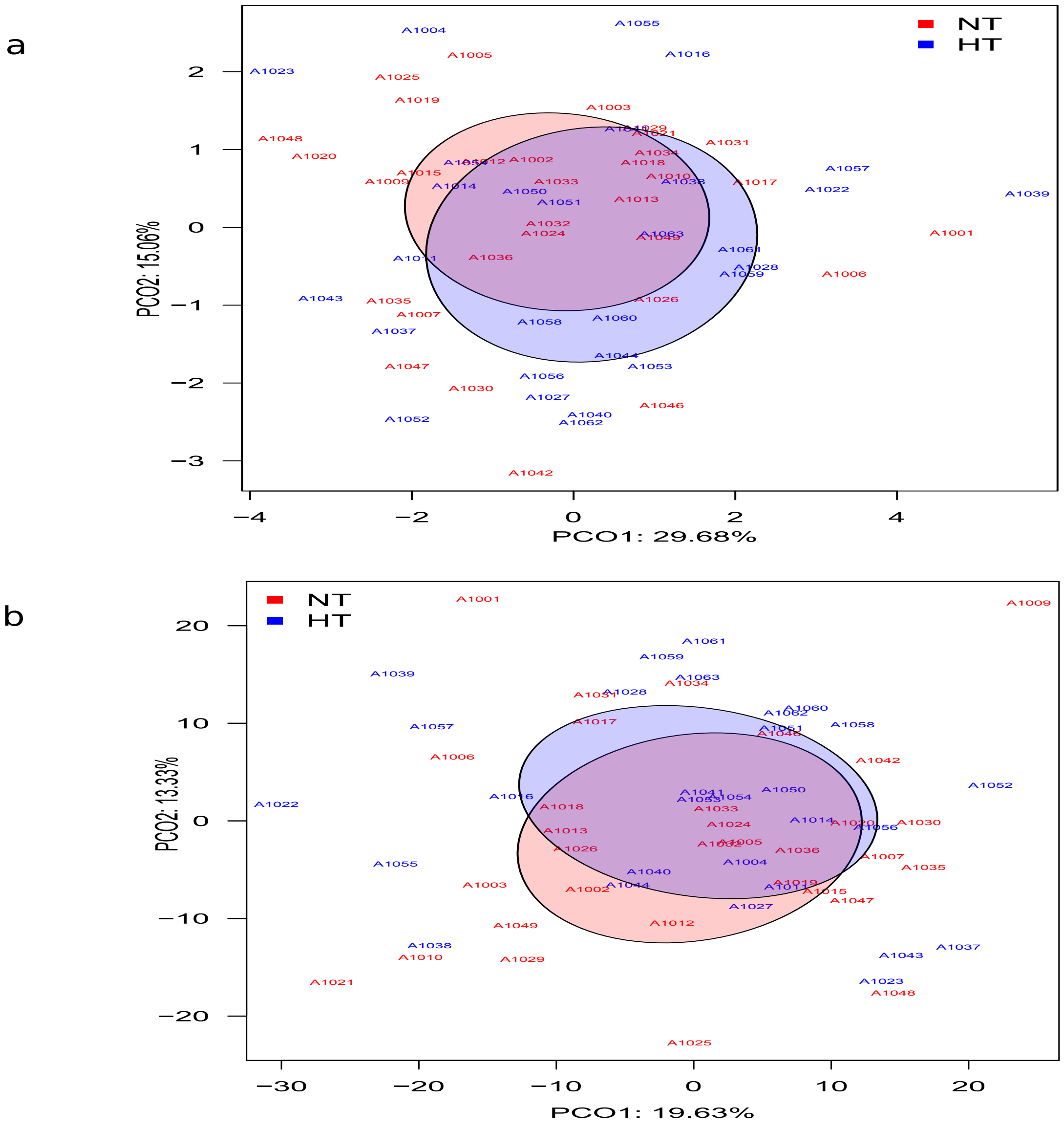

Additional file 5: Figure S3: Beta diversity. Principal Coordinates Analyses (PCoA) based on Bray-Curtis dissimilarity index at functional level between NT and HT groups. a Subrole level. b TIGRFAM level

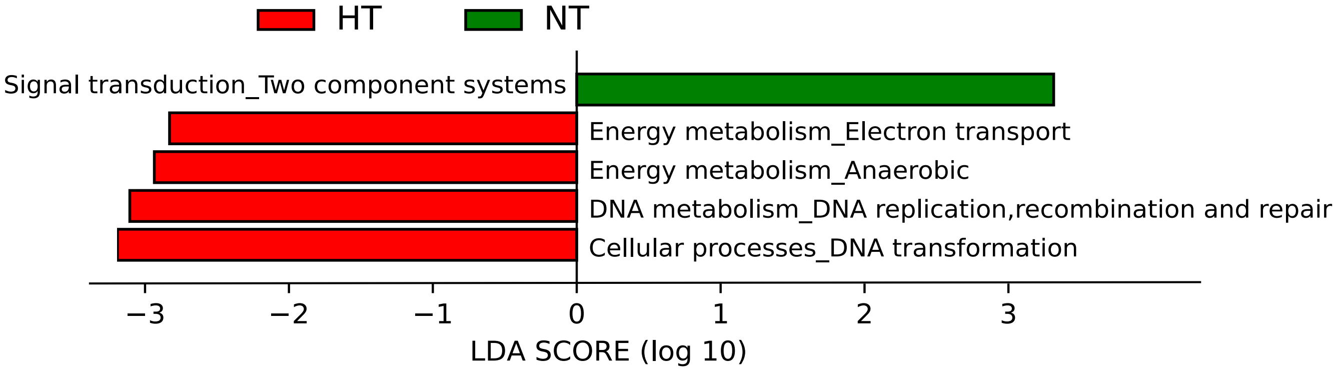

Additional file 6: Figure S4: LEfSe analysis of subroles between NT (green) and HT (red). LDA scores (log10) for the most prevalent subrole in NT group is represented in positive scale whereas LDA-negative scores indicated enriched subroles in HT group.

Additional file 7: Table S3 | Correlations between microbial metabolites (SCFA and TMAO) and clinical and dietary parameters.

	Acetate				Propionate				Butyrate				Valeric acid				Isobutyric acid				Isovaleric acid				TMAO	
	r^a	r^b	p^a	p^b	r^a	r^b	p^a	p^b	r^a	r^b	p^a	p^b	r^a	r^b	p^a	p^b	r^a	r^b	p^a	p^b	r^a	r^b	p^a	p^b	r^b	p^b
Age	0.28	-0.07	**0.028**	0.574	0.27	-0.01	**0.034**	0.969	0.21	-0.01	0.101	0.923	0.23	0.07	**0.056**	0.571	0.32	-0.17	**0.010**	0.198	0.23	-0.20	0.069	0.126	0.05	0.682
Gender	0.14	-0.16	0.279	0.213	-0.13	-0.10	0.302	0.453	-0.08	0.12	0.526	0.348	-0.12	0.15	**0.040**	0.248	-0.10	0.21	0.446	0.115	-0.12	0.14	0.347	0.300	0.10	0.438
Weight	0.29	-0.07	**0.020**	0.572	0.28	-0.04	**0.028**	0.766	0.28	-0.25	**0.025**	0.059	0.26	-0.17	**0.002**	0.205	0.29	-0.20	**0.021**	0.121	0.26	-0.10	**0.039**	0.465	-0.08	0.514
BMI	0.30	-0.29	**0.018**	**0.023**	0.31	-0.10	**0.013**	0.432	0.28	-0.23	**0.026**	0.079	0.25	-0.09	**0.006**	0.495	0.32	-0.15	**0.011**	0.255	0.25	-0.15	**0.050**	0.267	0.02	0.990
Waist circumference	0.34	-0.11	**0.009**	0.428	0.32	-0.06	**0.015**	0.667	0.26	-0.07	**0.055**	0.629	0.25	-0.10	**0.003**	0.482	0.33	-0.22	**0.011**	0.103	0.25	-0.21	0.058	0.131	-0.11	0.394
Fasting blood glucose	0.00	-0.27	0.995	**0.037**	0.02	-0.06	0.874	0.629	0.04	-0.16	0.754	0.232	0.00	-0.10	0.336	0.455	0.05	-0.08	0.722	0.557	0.00	-0.05	0.979	0.696	0.27	**0.034**
Total cholesterol	0.22	-0.01	0.089	0.915	0.19	0.13	0.153	0.320	0.21	-0.02	0.112	0.880	0.04	0.05	0.284	0.685	0.12	-0.10	0.360	0.429	0.04	0.03	0.761	0.845	0.28	**0.030**
HDL-cholesterol	0.15	-0.07	0.254	0.580	-0.22	-0.07	0.088	0.580	-0.11	-0.06	0.386	0.646	-0.06	0.04	0.101	0.751	-0.08	0.11	0.525	0.423	-0.06	0.04	0.652	0.742	-0.02	0.848
LDL-cholesterol	0.24	0.01	0.065	0.939	0.28	0.11	**0.029**	0.414	0.18	-0.13	0.159	0.333	0.15	-0.03	0.087	0.842	0.22	-0.19	0.086	0.140	0.15	-0.10	0.252	0.436	0.30	**0.019**
Triglycerides	0.20	-0.05	0.117	0.710	0.29	0.11	**0.020**	0.410	0.32	-0.02	**0.011**	0.850	-0.04	-0.05	0.083	0.711	0.02	-0.22	0.887	0.093	-0.04	-0.02	0.750	0.889	0.03	0.785
SBP	0.37	-0.25	**0.003**	0.055	0.35	-0.10	**0.005**	0.443	0.36	-0.23	**0.004**	0.072	0.06	-0.18	**0.019**	0.176	0.17	-0.42	0.179	**0.001**	0.06	-0.23	0.667	0.075	0.11	0.371
DBP	0.39	-0.26	**0.002**	**0.045**	0.35	-0.11	**0.005**	0.405	0.44	-0.23	**0.000**	0.073	0.04	-0.10	**0.006**	0.441	0.15	-0.33	0.255	**0.009**	0.04	-0.30	0.761	**0.017**	-0.01	0.964
Physical activity	0.09	0.01	0.490	0.963	0.08	0.02	0.516	0.903	-0.12	0.08	0.345	0.548	-0.24	0.11	0.293	0.405	-0.19	0.25	0.152	**0.047**	-0.24	0.14	0.068	0.290	0.07	0.591
Carbohydrates	-0.20	0.18	0.117	0.170	-0.20	0.28	0.107	**0.029**	-0.22	0.15	0.079	0.261	-0.32	0.29	**0.026**	0.023	-0.33	0.19	**0.009**	0.148	-0.24	0.12	0.064	0.356	0.01	0.971
Starches	-0.22	0.15	0.086	0.259	-0.23	0.29	0.064	**0.020**	-0.26	-0.01	**0.041**	0.940	-0.34	0.12	**0.020**	0.346	-0.38	0.11	**0.002**	0.416	-0.29	0.12	**0.022**	0.365	0.08	0.507
Fats	-0.19	0.14	0.125	0.270	-0.20	-0.03	0.106	0.807	-0.20	0.04	0.119	0.785	-0.12	0.04	0.328	0.780	-0.17	0.14	0.200	0.285	-0.09	0.08	0.500	0.563	0.10	0.446
Protein	-0.15	0.11	0.221	0.399	-0.22	0.05	0.083	0.714	0.13	-0.05	0.204	0.704	-0.14	0.01	0.279	0.922	-0.20	0.15	0.130	0.262	-0.09	0.22	0.468	0.086	0.15	0.253
Dietary fiber	-0.18	0.25	0.151	0.058	-0.26	0.18	**0.038**	0.173	-0.21	0.08	0.103	0.522	-0.26	0.21	**0.015**	0.102	-0.25	0.20	**0.044**	0.128	-0.17	0.13	0.201	0.316	0.46	0.098
Ethanol	0.46	0.06	0.726	0.675	0.02	-0.02	0.865	0.857	0.03	-0.18	0.777	0.173	0.12	-0.24	0.330	0.059	0.25	-0.27	**0.044**	**0.037**	0.25	-0.18	**0.048**	0.179	0.32	0.808

BMI, body mass index; LDL, low density lipoproteins; HDL, high density lipoproteins; SBP, systolic blood pressure; DBP, diastolic blood pressure. Significant correlations depicted in **bold**. N=61

r^a: Pearson correlation coefficient between faecal SCFA and clinical and dietary variables; r^b: Pearson correlation coefficient between plasma SCFA and TMAO and clinical and dietary variables; p^a: Test of significance for faecal SCFA correlations; p^b: Test of significance for plasma SCFA and TMAO correlations

Relationship of SCFA concentrations with SBP and DBP. r. Pearson correlation coefficient and p-value (n=61)

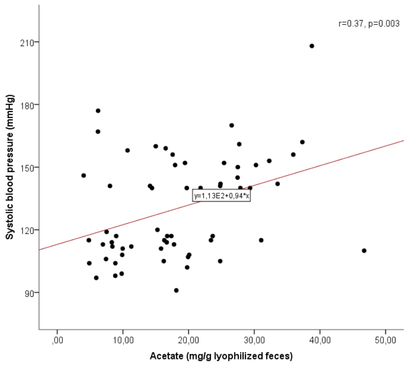

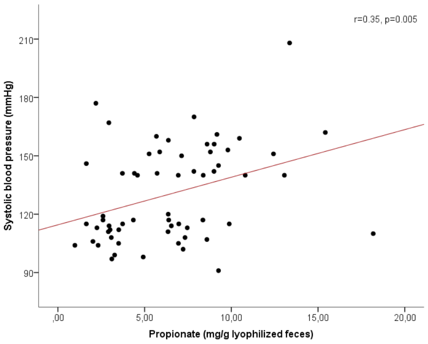

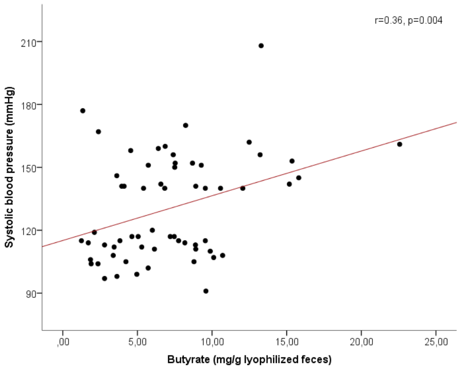

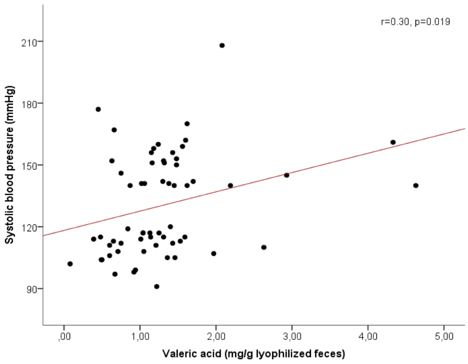

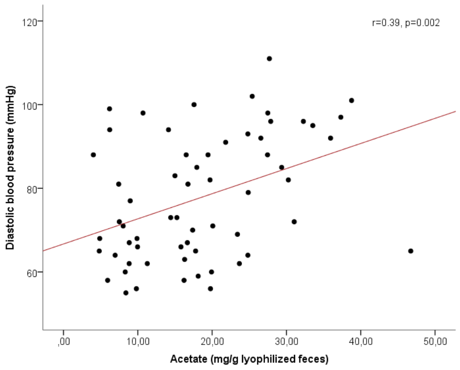

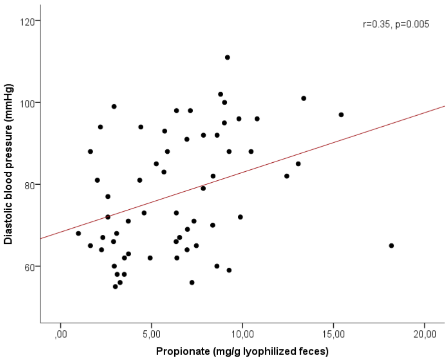

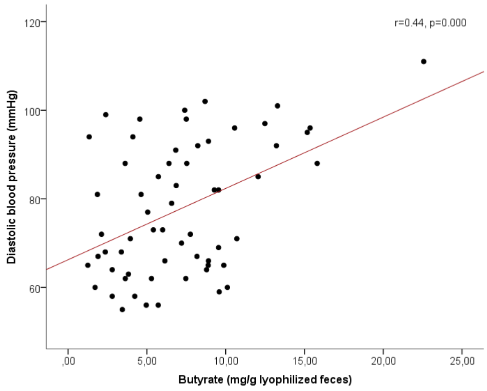

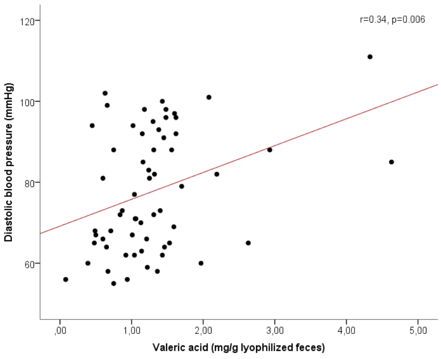

Supplement: Supplementary file 1 — Supplementary information. [file 41598_2020_63475_MOESM1_ESM.pdf]
